# Annotations

**Published:** 1896-01-25

**Authors:** 


### ANNOTATIONS.

### The Whitechapel Guardians and the Local Doctors.

THE public life of East London is apt, not merely to sharpen the wits of its administrators, but also to make those gentlemen considerate and reasonable in their general behaviour. A satisfactory proof of this is furnished by the new departure in relation to extra medical services published in the annual report of the Whitechapel Guardians, which has just been issued. A sensible coroner, moved no doubt by his experience of the delays which so often occur in procuring immediate medical aid in cases of dire emergency among the very poor, made the practical suggestion that the really necessitous poor should, in cases of emergency, be empowered by the guardians to call in the nearest medical aid for one visit without the preliminary of procuring a relieving officer's order; and that for the one urgent visit, and the medicine which might be required, the guardians themselves should pay. The clerk to the Whitechapel Board, as kindly disposed as the coroner, made known the suggestion to the guardians, and they in their turn sought the advice and authority of the Local Government Board on the subject. The Local Government Board decided that by Sec. 2 of the 11 and 12 Vict., cap. 110, the guardians are empowered "to pay for any medical assistance rendered to any poor person by reason of accident, bodily casualty, or sudden illness, although no order shall have been given by them, or their officers, or the overseers." Acting upon this the Whitechapel Guardians have issued a circular to the medical practitioners within the union to the effect that they may, if called upon, in the case of the urgent sickness of a destitute person, make one visit and supply the necessary medicine, and that for such visit the guardians will pay them at the rate of 7s. 6d. if made in the night, and of 3s. 6d. if made in the daytime. A practical solution has thus been made of a practical difficulty. Our reason for calling attention to the new departure is that this very subject has been a *quæstio vexata* between the medical profession and boards of guardians in many places for many years. To our thinking the Whitechapel Guardians have terminated the difficulty in the only reasonable and equitable way. Perhaps we may be permitted to say to all boards of guardians which have not yet considered the question, "Go ye and do likewise."

### The Evolution of the Winning Races.

THE modern evolutionist, not before time, has begun to study a phenomenon which his predecessors were inclined to despise—the significant phenomenon that practically all men everywhere are religious. Now, whether we be religionists, agnostics, or atheists, this one thing is certain, that religion is a universal fact in history and experience. To attempt, therefore, to construct a philosophy of man, and to ignore his religion throughout, is about as childish a performance as to attempt to explain the solar system, leaving out all reference to the sun. Mr. Benjamin Kidd, one of our most recent and most able writers on "Evolution," tells us that "the winning races have always been those in which, other things being equal, the religious character has been most fully developed." And "amongst these again," he continues, "the races that have acquired an ever-increasing ascendancy have been those which have possessed the best ethical systems." Well, this is so, or it is not. Some persons, who call themselves students of sociology, will dismiss the question without further ado as unworthy of serious consideration. It is quite certain that, however much such persons may write they will never throw much real light upon questions of social science, simply because they write with half-closed eyes. The facts are the facts. Science must deal with the facts, or she will prove herself absolutely sterile. The days of prejudice and preconceived speculation are over. The world may be a fool; but, if it be, it is a religious fool; and we cannot do better than remind scientific persons of the fact, and ask them to look at it afresh, and that with good humour. It is, we repeat, either true or not true that the winning races are the religious and the ethical. If it be true, science will not do a good turn either to the world or to herself by persistently ignoring the factor of religion in her discussion of the problems of social evolution. In short, that evolutionary science is no science at all which does not recognise, explain, and estimate the true value of all the facts that go to make up the whole case.

### Milk as a Vehicle of Disease.

IN a recent lecture before the National Health Society Dr. Thorne Thorne showed that the principal diseases of which milk was now known to act as the vehicle were typhoid fever, cholera, scarlet fever, diphtheria, and tuberculosis; but he drew a wide distinction between the manner in which it carried the infection in these different diseases, a distinction which is of some importance in searching out the origin of epidemics in the spread of which milk has clearly been concerned. Both typhoid fever and cholera may be looked upon as maladies which result from fouling of the water supply, and milk acts as a vehicle of these ailments just in proportion as it becomes mixed in any way with specifically polluted water. Neither of these diseases is ever carried by milk except from an antecedent case in man. The milk is, in fact, but a carrier of the infection from man to man. In scarlet fever, diphtheria, and tuberculosis, however, the matter is different. The cows themselves are affected with a disease, and as a result they distribute the infection in their milk. It may be quite impossible to discover any cases of the disease in question among people who have had to do with the milk in its collection or distribution, and it would appear by no means certain that these are not really animal diseases implanted upon man. At any rate, what clearly comes out is this, that while for the prevention of the spread of typhoid and cholera protection of the milk from infection is sufficient, the prevention of scarlet fever, diphtheria, and tuberculosis demands not only supervision of the milk, but of the cows by whom it is produced. Till measures for this purpose can be enforced, the only safety lies in boiling the milk, the necessity for doing which few would hesitate to admit did they once realise that in England and Wales upwards of 60,000 persons per annum die from tuberculous diseases alone.

